# Generalized Concept and MATLAB Code for Modeling and Analyzing Wideband 90° Stub-Loaded Phase Shifters with Simulation and Experimental Verifications

**DOI:** 10.3390/s23187773

**Published:** 2023-09-09

**Authors:** Falih M. Alnahwi, Yasir I. A. Al-Yasir, Chan Hwang See, Abdulkareem S. Abdullah, Raed A. Abd-Alhameed

**Affiliations:** 1 Department of Electrical Engineering, College of Engineering, University of Basrah, Basrah 61001, Iraq; falih.mousa@uobasrah.edu.iq (F.M.A.); abdulkareem.abdullah@uobasrah.edu.iq (A.S.A.); 2 Faculty of Engineering and Informatics, University of Bradford, Bradford BD7 1DP, UK; r.a.a.abd@bradford.ac.uk; 3 School of Computing, Engineering and the Built Environment, Edinburgh Napier University, Edinburgh EH10 5DT, UK; c.see@napier.ac.uk; 4 Department of Information and Communication Engineering, Basrah University College of Science and Technology, Basra 61004, Iraq

**Keywords:** phase shifter, stub-loaded filter, reflection coefficient, transmission coefficient, differential phase

## Abstract

In the design of phase shifters, the modeling equations are too complicated and require some approximations to be derived correctly by hand. In response to this problem, this paper presents a generalized concept, algorithm, and MATLAB code that provide the exact modeling equations of the transmission parameters and the scattering parameters of any 90° wideband stub-loaded phase shifter. The proposed code gives the modeling equations in term of variables for any number of stubs and characteristic impedance value by utilizing the symbol-based analysis of the MATLAB code. It also illustrates the results as a function of normalized frequency relative to the center frequency *f_o_*, and can be and can be tailored to any user-defined frequency range. As a matter of comparison, a three-stub wideband 90° stub-loaded phase shifter is simulated using CST Microwave Studio and experimentally fabricated on Rogers RT5880 dielectric substrate with dimensions of 30 × 40 × 0.8 mm^3^. The comparison reveals the accuracy of the proposed computerized modeling with −10 dB impedance bandwidth equal to 90% (0.55*f_o_*–1.45*f_o_*), (90°∓5°) phase difference bandwidth equal to 100% (0.5*f_o_*–1.5*f_o_*), and negligible insertion loss. The novelty of this work is that the proposed code provides the exact modeling equations of the stub-loaded phase shifter for any number of stubs regardless the complexity of the mathematical derivations.

## 1. Introduction

90° microwave phase shifters are devices that are widely used in variety of industrial applications. These phase shifters are commonly used in 5G and 6G beamforming systems [1,2,3,4]. The 90° phase shifters are also indispensable in exciting narrowband circularly polarized antennas [5] and broadband circularly polarized antennas [6,7]. In addition to these well-known applications, the utilization of the 90° microwave phase shifter extends to the design of balanced mixers, modulators, beam scanning, and so on. Most of the mentioned applications are broadband, so the phase shifter that is used in these kinds of systems should perfectly cover the bandwidth of these devices. Therefore, many researchers have oriented their focus toward the design of planar wideband phase shifters for their low dispersive propagation properties [8,9]. However, for each design procedure the mathematical model is presented to provide a mathematical verification that support the outcomes of each design.

An ultra-wideband phase shifter was designed in [8] using a slot coupling double-layer structure, and it was analyzed using the conventional coupled-line analysis. In [10], wideband phase shifting was achieved using multi-mode resonator with cascade line sections, and the conventional transmission matrix analysis was used in deriving the scattering parameters with some approximations. A stub-loaded multi-mode resonator was coupled with the input and output lines of a phase shifter to provide filtered phase shifting with reduced out-of-band interference. A balanced wideband phase shifter was proposed in [11], and it was analyzed using the conventional differential and common mode analysis. The design in [12] is a 90° sub-loaded phase shifter with three pen circuit stubs. The design applied some approximations in driving the transmission and scattering parameters of the proposed system. Even and odd mode analysis [13,14] was applied on a simple and compact coupled-line phase shifter to study the phase linearity of the structure. In [15], a cascade wideband phase shifter and a band stop network were combined to form dual-band phase shifter. The design was modeled using the cascade analysis of the transmission matrices of each design. Slot lines were used in [16] to design a quasi-Schiffman phase shifter. A reconfigurable phase shifter was designed in [17] with the aid of two PIN diodes. Some phase shifters were attached directly to the antenna to provide a radiation with inherent phase shifting [18]. The authors in [19] considered the strong coupling phenomena between the phase shifters and the nearby components.

All the aforementioned research either avoids the mathematical modeling or uses the conventional mathematical derivations with some approximations to simplify the analysis. In this work, a generalized algorithm and MATLAB code are proposed to derive the exact modeling equations of N-stub wideband 90° stub-loaded phase shifter with high accuracy. The proposed modeling equations are deal with the characteristic impedances, electrical length, and the normalized frequency, and can be specified for any numerical values. The rest of the paper is organized as follows: Section 2 explains the basic concept that should be followed in generating wideband phase shifting, while Section 3 gives the general modeling steps of the stub-loaded phase shifter. In Section 4, the generalized algorithm and the MATLAB code that are proposed in this paper are demonstrated and discussed in detail. A parametric study for the proposed model for different characteristic impedance values and different numbers of stubs is presented in Section 5. Section 6 compares the outcomes of the proposed modeling equations with simulated and measured results of three-stub wideband 90° phase shifter. Finally, Section 7 summarizes the whole paper in a brief conclusion.

## 2. Concept of Wideband Differential Phase Shifting

**Figure 1:**
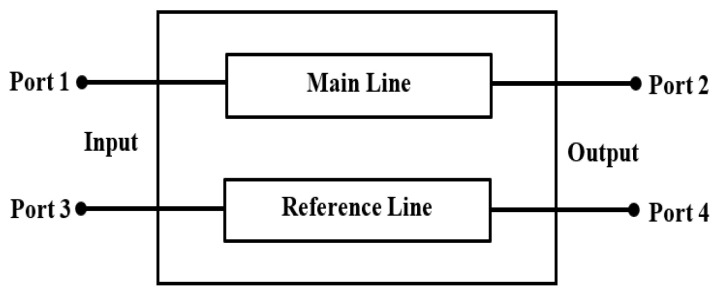
Differential phase shifter as a four-port network.

The differential phase shifter is a four-port network with two input ports and two output ports. Figure 1 shows the phase shifter as a four-port network, considering that Port 1 and 3 are the input ports, while Port 2 and 4 are the output ports. The reference line is a conventional transmission line, whereas the main line is designed to provide a specific phase shift between the signals of Port 2 and Port 4 over a wide range of frequencies with almost equal amplitudes. From a mathematical point of view, the difference between the phase angle of the transmission coefficient $S_{21}$and that of the transmission coefficient $S_{43}$ should be fixed over a wide range of frequencies, such as:

$$∠S_{21}-∠S_{43}=constant$$

(1) $$\left|S_{21}\right|≅\left|S_{43}\right|\;over\;a\;wide\;range\;of\;frequencies$$

As given in [20], the transmission parameter of the lossless transmission line (reference line) is given by:

(2) $$S_{43}=e^{-j\theta_{ref}}$$

(3) $$\theta_{ref}=\beta l_{ref}$$

(4) $$\beta =\frac{2\pi }{\lambda }=\frac{2\pi }{c}f$$

where $\theta_{ref}$ represents the electrical length of the reference line, $l_{ref}$ denotes the length of the reference line, β represents the phase constant of the reference line, λ is the wavelength, f represents the frequency, and c is the speed of the line in free space (3 × 10^8^ m/s). By substituting (3) and (4) into (2), the phase angle of the transmission coefficient can be given as in (5):

(5) $$∠S_{43}=-\frac{2\pi l_{ref}}{c}f$$

It is clear that the phase shift of the reference line is a linear equation with respect to the frequency. On the other hand, the main line is a two-port network that results a complex transmission parameter $S_{21},$with phase angle given by (6):

(6) $$∠S_{21}=\tan^{-1}\left(\frac{Imaginary\left(S_{21}\right)}{Real\left(S_{21}\right)}\right)$$

**Figure 2:**
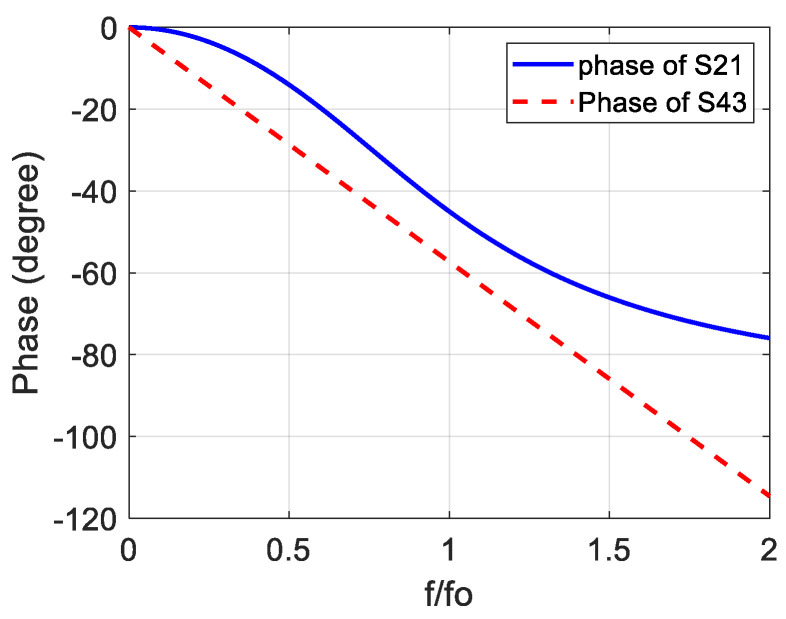
Hypothetical phases of the main line ($∠S_{21}$) and the reference line.

Figure 2 demonstrates the hypothetical phase of the main line ($∠S_{21}$) and the hypothetical phase of the reference line ($∠S_{43}$) as a function of the normalized frequency ($f/f_{o}$), where $f_{o}$ represents the center frequency of the phase shifter. It is known that the inverse tangent function has two saturation regions and a quasi-linear region in the middle of them. Therefore, two main considerations can be taken into account to obtain wideband constant differential phase shift:

a. The linear region of the phase of the main line ($∠S_{21}$) should be as wide as possible with respect to the frequency.
b. The linear region of $∠S_{21}$ should be parallel with the phase of the reference line ($∠S_{43}$) to provide constant phase difference along the frequency range of the linear region.

## 3. Analysis of Stub-Loaded Wideband Phase Shifter

**Figure 3:**
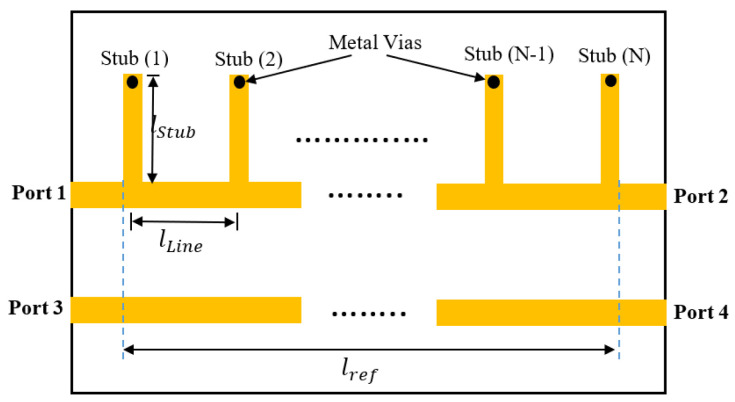
Design steps of the proposed wide-slot antenna.

Notably, the phase response of the stub-loaded transmission line has a wideband linear region. Consequently, it can be used as a main line in the wideband phase shifter, as mentioned in the previous section. The general structure of the stub-loaded phase shifter with N parallel stubs is illustrated in Figure 3. the stub-loaded line has a band-pass characteristics with almost linear phase within its pass band [20]. Each stub is short circuited to the ground plane with the aid of metal vias.

The length of the stub $l_{Stub}$ and the length of the line section between every two consecutive stubs $l_{Line}$ are selected to be equal (1/4) to the guided wavelength $\lambda_{go}$ of the center frequency ($f_{o}$). The characteristic impedance of the reference line and the main line is $Z_{o}$, while the characteristic impedance of the parallel stubs is $Z_{p}$. The transmission matrices of the line sections $T_{Line}$ and the short circuit stub $T_{Stub}$ are given by [20]:

(7) $$T_{Line}=\left[\begin{array}{cc} \cos\left(\theta_{1}\right) & jZ_{o}\sin\left(\theta_{1}\right)\\ \frac{j}{Z_{o}}\sin\left(\theta_{1}\right) & \cos\left(\theta_{1}\right) \end{array}\right]$$

(8) $$T_{Stub}=\left[\begin{array}{cc} 1 & 0\\ \frac{-j}{Z_{p}}\cot\left(\theta_{2}\right) & 1 \end{array}\right]$$

where $\theta_{1}$ and $\theta_{2}$ are the electrical length of the line sections and the stub, respectively. It is clear that the general structure of the stub-loaded line consists of (N) parallel stubs and $\left(N-1\right)$ line sections connected in cascade. Therefore, the overall transmission matrix T of the stub-loaded line can be found by multiplying the N transmission matrices of the stubs by the $\left(N-1\right)$ transmission matrices of the line sections, alternately, as follows:

(9) $$T=\left[\begin{array}{cc} A & B\\ C & D \end{array}\right]=T_{Stub\left(1\right)}T_{Line\left(1\right)}T_{Stub\left(2\right)}\ldots T_{Line\left(N-1\right)}T_{Stub\left(N\right)}$$

After deriving the overall transmission matrix, the scattering matrix of the stub-loaded line can easily be obtained. Since the stub-loaded line is a symmetric and reciprocal two port network, the scattering parameters can be found as follows [20]:

(10) $$S_{11}=S_{22}=\frac{\left(A-D\right)+\left(\frac{B}{Z_{o}}-CZ_{o}\right)}{\left(A+D\right)+\left(\frac{B}{Z_{o}}+CZ_{o}\right)}$$

(11) $$S_{21}=S_{12}=\frac{2}{\left(A+D\right)+\left(\frac{B}{Z_{o}}+CZ_{o}\right)}$$

The above derivation requires very intensive mathematics and accurate analysis to obtain the correct equations for the reflection coefficient $S_{11}$and the transmission coefficient $S_{21}$, so we have proposed a MATLAB code that accurately derives the above equations. In addition, the code can also be specified for any design constraints selected by the user.

## 4. General Code for Modelling 90° Stub-Loaded Phase Shifter

The download link of the MATLAB code presented in this section is given in Appendix A at the end of the paper. This code is composed of two parts. The first part deals with deriving the transmission matrix and the scattering matrix of the stub-loaded line after selecting its number of stubs. On the other hand, the second part of the MATLAB code demonstrates the scattering parameters and the resulting differential phase shifting as a function of the normalized frequency ($f/f_{o})$ for certain values of $Z_{o}$ and $Z_{p}$.

### 4.1. Part 1: Deriving the T and S Matrices

This part of the proposed MATLAB code starts with selecting the number of stubs (N) of the stub-loaded line. The symbol-based analysis in MATLAB is achieved by using the MATLAB function “syms”, which holds $Z_{o}$, $Z_{p}$, $\theta_{1}$, and $\theta_{2}$. The algorithm that is then used to obtain the T and S matrices is given below:

1. Enter the value of the number of steps N, then the symbols $Z_{o}$, $Z_{p}$, $\theta_{1}$, and $\theta_{2}$ are defined using the “syms” MATLAB function.
2. Write the equations of $T_{Line}$ and $T_{Stub}$, given in (7) and (8).
3. Initialize the transmission matrix of the stub-loaded filter by $T=T_{Stub}$.
4. Multiply the previous value of T by ($T_{Line}\times T_{Stub}$).
5. Repeat Step (4) (N−1) times to obtain a multiplication of N times $T_{Stub}$ by (N−1) times $T_{Line}$, alternately, as given in (9).
6. Use the MATLAB functions “expand” and “simplify” for simplifying the results and finding some simple trigonometric identities.
7. Apply (10) and (11) to find the reflection and transmission parameters of the S-matrix.

As an example for the above steps, the code is executed for N=3. The ABCD parameters of the T-Matrix for N=3 resulted from the MATLAB code are:

(12a) $$A={cos}^{2}\left(\theta_{1}\right)-{sin}^{2}\left(\theta_{1}\right)+{\left(\frac{Z_{o}}{Z_{p}}\right)}^{2}{cot}^{2}\left(\theta_{2}\right){sin}^{2}\left(\theta_{1}\right)+3\left(\frac{Z_{o}}{Z_{p}}\right)cos\left(\theta_{1}\right)sin\left(\theta_{1}\right)cot\left(\theta_{2}\right)$$

(12b) $$B=j\left(2Z_{o}cos\left(\theta_{1}\right)sin\left(\theta_{1}\right)+\frac{Z_{o}^{2}}{Z_{p}}cot\left(\theta_{2}\right){sin}^{2}\left(\theta_{1}\right)\right)$$

(12c) $$C=-j\left(\frac{1}{Z_{p}}{cos}^{2}\left(\theta_{1}\right)+\frac{Z_{o}^{2}}{Z_{p}^{3}}{cot}^{3}\left(\theta_{2}\right){sin}^{2}\left(\theta_{1}\right)+\frac{2}{Z_{p}}cot\left(\theta_{2}\right)\left({cos}^{2}\left(\theta_{1}\right)-{sin}^{2}\left(\theta_{1}\right)\right)+2\left(\frac{2Z_{o}}{Y_{p}^{2}}cot\left(\theta_{2}\right)-\frac{1}{Z_{o}}\right)sin\left(\theta_{1}\right)cos\left(\theta_{1}\right)\right)$$

(12d) D=A

It is clear that the Equations (12a)–(12d) are too complicated and very eligible for mistakes using derivation by hand. In addition, increasing the number of stubs results in more complicated equations. For this reason, many researchers have used some approximations to simplify the derivation, but some tolerance in the outcomes of the model still appears. However, the proposed code gives the exact modeling equations since the derivation is computerized in this work.

### 4.2. Part 2: Specified Variable Results

After executing its first part, the code asks the user to inter certain values for $Z_{o}$ and $Z_{p}$. Before delving in the proposed algorithm, it is important to write the arbitrary electrical length (θ) in term of the normalized frequency ($f/f_{o}$) and the normalized length of an arbitrary line ($l_{norm}=l/\lambda_{o}$), where l is the line length and $\lambda_{o}$ is the wavelength of the center frequency $f_{o}$. By substituting (4) into the electrical length (θ=βl), then putting ($l=l_{norm}\lambda_{o}$), and substituting the well-known formula ($\lambda_{o}=c/f_{o}$), the electrical length can be re-written in terms of the normalized frequency:

This equation is valid for calculating $\theta_{1}$, $\theta_{2}$, and $\theta_{ref}$ in term of the normalized lengths $l_{Line-norm}$, $l_{Stub-norm}$, and $l_{ref-norm}$, respectively. One should note that it is important to use the MATLAB function “phase” and not the function “angle” in calculating the phase angle of any parameter, because the function “angle” results in phase angle wrapped between −180° and 180°.

The algorithm that describes the second part of the proposed code is as follows:

1. Enter values for $Z_{o}$ and $Z_{p}$, so that the best impedance bandwidth and phase difference bandwidth is achieved.
2. Set the variable normalized frequency to any range (say 0 to 2 with 0.01 step size).
3. Set the normalized lengths of the stub and the line sections to 0.25 which is corresponding to quarter wavelength line at the center frequency.
4. In the resulted $S_{11}$ and $S_{21}$, use the MATLAB function “subs” at each value of the normalized frequency to substitute the values of $Z_{o}$ and $Z_{p}$. In addition, substitute the value of $\theta_{1}$, $\theta_{2}$with the aid of (12).
5. Find the magnitude of $S_{11}$ and $S_{21}$ in dB, as well as the phase of $S_{21}$ in degrees using the MATLAB function “phase”.
6. The normalized length of the reference line is equal to the length of (N−1) line section plus 0.25 to provide 90° phase delay at $f_{o}$ with respect to the main line.
  (13) $$l_{ref-norm}=\left(N-1\right)l_{Line-norm}+0.25$$
7. Find the phase of transmission coefficient $S_{43}$of the reference line, which is equal to
  (14) $$-\theta_{ref}=-2\pi l_{ref-norm}(f/f_{o})$$
8. The phase difference at the output ports is equal to ($∠S_{21}-∠S_{43}$).

As mentioned earlier, the MATLAB code consisting of the two parts followed by some figure demonstrations is given in Appendix A.

## 5. Parametric Study for the Proposed Modelling

**Figure 4:**
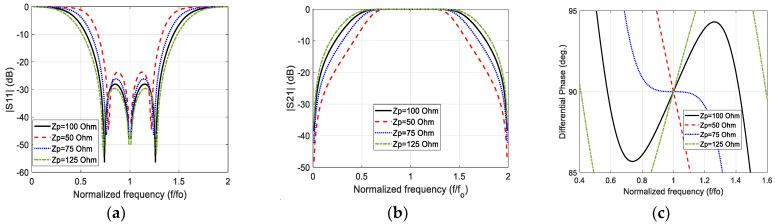
The modeled (**a**) reflection coefficient magnitude; (**b**) transmission coefficient magnitude; and (**c**) phase difference between the output ports for N=3, $Z_{o}=50\;\Omega$, and different values of $Z_{p}$.

It is important to make a parametric study for the proposed model to find the suitable value of the stub characteristic impedance that results in as wide an impedance bandwidth as possible and as wide a phase difference bandwidth as possible. Figure 4 illustrates the modeled magnitude of the reflection coefficient, the magnitude of the transmission coefficient, and phase difference of three-stub (N=3) wideband 90° stub-loaded phase shifter for different stub characteristic impedance values and $Z_{o}=50\;\Omega$. It is found that at $Z_{p}=100\;\Omega$, the −10 dB impedance bandwidth covers the normalized frequency range $0.55f_{o}–1.45f_{o}$ (90%), and $\left(90{}^{\circ}\mp 5{}^{\circ}\right)$ phase difference bandwidth equal to 100% covering the range $0.5f_{o}–1.5f_{o}$. The impedance bandwidth at this value of impedance is perfectly covered by the phase difference bandwidth with constant transmission coefficient close to zero dB, which verifies the negligible insertion loss of the proposed main line within the operational band.

**Figure 5:**
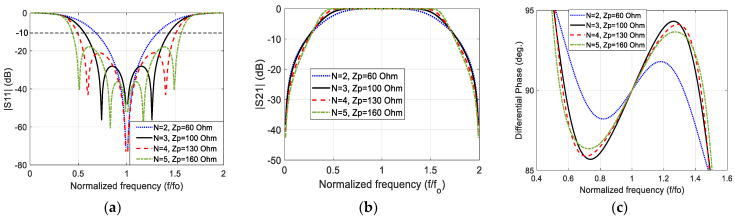
The modeled (**a**) reflection coefficient magnitude; (**b**) transmission coefficient magnitude; and (**c**) phase difference between the output ports for $Z_{o}=50\;\Omega$ and different values of N and $Z_{p}$.

Using the same parametric study, the convenient stub characteristic impedance that results in the widest impedance bandwidth and widest phase difference bandwidth for *N* = 2, 4, and 5 are equal to 60 Ω, 130 Ω, and 160 Ω, respectively. Figure 5 exhibits the modeled magnitude of the reflection coefficient, the magnitude of the transmission coefficient, and the phase difference of the stub-loaded wideband 90° phase shifter for different stub characteristic impedance values and *Z_o_* = 50 Ω and different number of stubs.

It is clear that increasing the number of stubs results in a noticeable improvement in the −10 dB impedance bandwidth and the flatness of the pass-band of the stub-loaded line (main line). However, the improvement in the $\left(90{}^{\circ}\mp 5{}^{\circ}\right)$ phase difference bandwidth is insensible. It is known that the width of the microstrip line decreases as the characteristic impedance increases. Since increasing the number of stubs requires higher characteristic impedance to provide $\left(90{}^{\circ}\mp 5{}^{\circ}\right)$ wider phase difference bandwidth, (N=3) is selected in this work to be compared with the standard CST Microwave Studio results and the experimental results. In other words, three-stub 90° stub-loaded phase shifter is used to verify the precision of this modeling because it results in reasonable stub width that can easily be fabricated by any PCB engraving machine. In addition, a large number of stubs requires larger dimensions than the smaller number of stubs.

**Figure 6:**
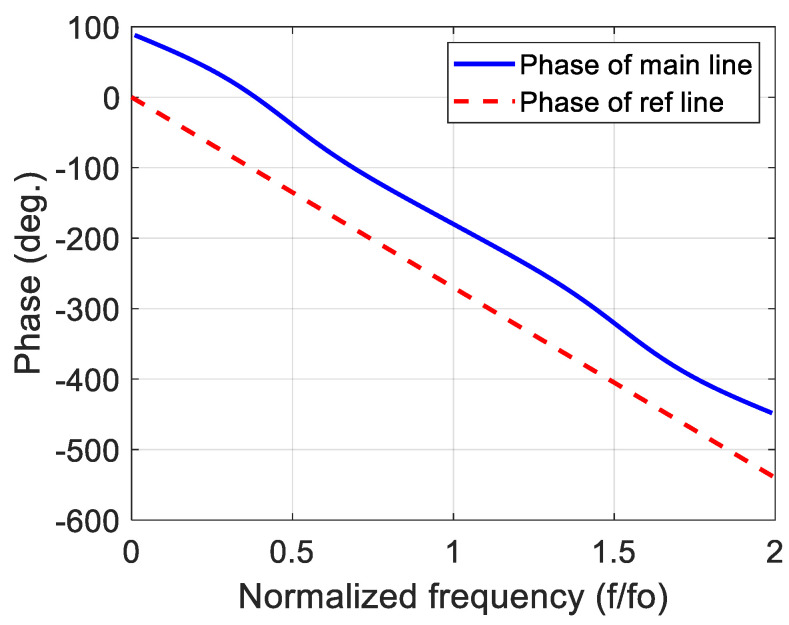
Phase responses of the main and the reference lines for *N* = 3 stub-loaded 90° phase shifter.

To fortify the claim of providing wideband phase shifting presented in Section 2, the modeled phase response of $S_{21}$of the 90° stub-loaded phase shifter with N=3 along with the phase response of $S_{43}$ is illustrated in Figure 6. The linear region of the phase of the $S_{21}$ can clearly be seen in this figure, and it is parallel to the phase of the $S_{43}$ with phase difference approximately equal to 90° at each frequency component.

## 6. Simulation and Experimental Verification Example

**Figure 7:**
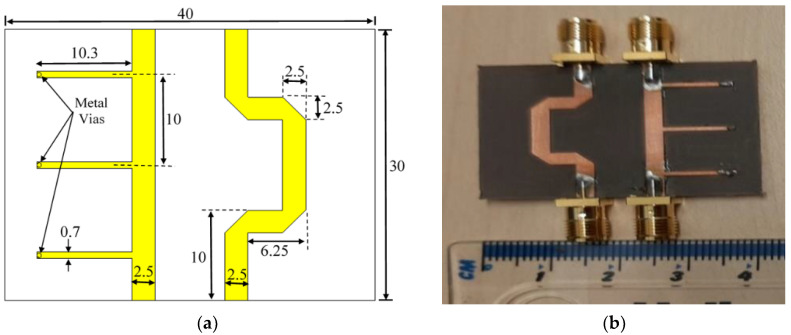
Three-stub 90° stub-loaded phase shifter: (**a**) CST microwave studio structure; (**b**) the prototype of the proposed design.

In this section, the proposed model is compared with its CST microwave studio equivalent, as well as the fabrication version of the design to verify the accuracy of the proposed MATLAB-based modeling equations. Figure 7a illustrates the dimensions of the CST simulated design, whereas Figure 7b exhibits the prototype of the proposed design. The center frequency of the design is selected to be *f_o_* = 5.5 GHz. The dielectric substrate of the proposed structure is Rogers RT5880, whose dielectric constant $\varepsilon_{r}=2.2$, height h=0.8 mm, with a loss tangent of 0.0009. The length of the stubs and the line sections are equal to a quarter of the guided wavelength ($\lambda_{go}$), corresponding to the center frequency $f_{o}$, which is equal to:

(15) $$\lambda_{go}=\frac{\lambda_{o}}{\sqrt{\varepsilon_{re}}}$$

where $\lambda_{o}$ represents the freespace wavelength of the center frequency and $\varepsilon_{re}$ is the effective dielectric constant that can be calculated from the well-known empirical equations given in [21,22]. The width of each transmission line is also calculated in term of $\varepsilon_{re}$ using the formulas in [21,22]. It is worth mentioning that the measurements were acquired using Agilent N5242A Vector Network Analyzer at the University of Bradford/Faculty of Engineering and Informatics.

**Figure 8:**
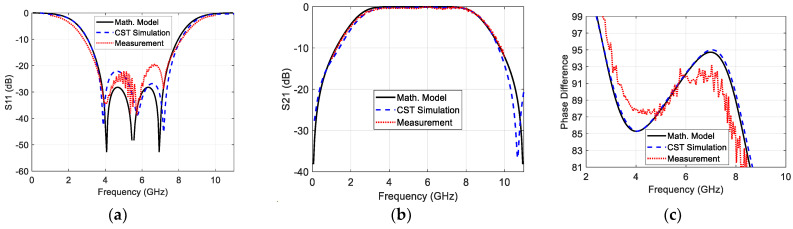
Comparison between the modeled, simulated, and measured (**a**) reflection coefficient magnitude; (**b**) transmission coefficient magnitude; and (**c**) phase difference between the output ports.

Figure 8 demonstrates a comparison between the outcomes of the proposed MATLAB-based modeling equations and the verification simulated and measured results for $f_{o}=5.5\;\mathrm{GHz}$. It is clear that the results of the modeled equations accurately aligned with the simulated and measured results. The −10 dB bandwidth is found to be about 90% (3.1–8 GHz) and $\left(90{}^{\circ}\mp 5{}^{\circ}\right)$ the phase difference bandwidth equal to 100% (2.8–8.3 GHz). Table 1 lists the −10 dB impedance bandwidth, the insertion loss, the $\left(90{}^{\circ}\mp 5{}^{\circ}\right)$ phase difference bandwidth of the modeled equations, and the standard simulation and measured results. The small deviation between the measured and simulation results is due to the imperfect SMA and vias soldering, the small approximations in calculating the dimensions of each piece of transmission line, and the irregular variation of the dielectric constant along the frequency.

One important thing should be noted. This work does not consider the strong coupling effect in the modeling of the proposed phase shifter. The strong coupling effect is deeply explained in [19]. As a future work, the proposed MATLAB code will consider the influence of the strong coupling to fortify the knowledge of this work.

It is important to note that the simulated and measure phase angle of any scattering parameter (∠S) are wrapped within the range [−180°–180°]. Therefore, they should be unwrapped before calculating the phase difference by constructing the complex values of the scattering parameter ($S=\left|S\right|e^{j∠S}$) and then calculating the phase of the resulted complex variable using the MATAB function “phase”. In [23], a coefficient called (The Effective Circular Polarization Percentage with Respect to the Antenna Bandwidth) is proposed to quantify the extension of the circular polarization over the antenna impedance bandwidth. This coefficient can be utilized in this work to quantify the extension of the $\left(90{}^{\circ}\mp 5{}^{\circ}\right)$ phase difference bandwidth over the −10 dB impedance bandwidth. This coefficient can be renamed to (The Effective Phase Difference Bandwidth Percentage with Respect to the Impedance Bandwidth (EPDBW%)):

(16) $$EPDBW\%=\frac{\left(-10\;\mathrm{dB}BW\right)\cap \left(PhaseDifferenceBW\right)}{-10\;\mathrm{dB}BW}\times 100\%$$

In this work, the EPDBW%=100% since the $\left(90{}^{\circ}\mp 5{}^{\circ}\right)$ phase difference bandwidth entirely covers the −10 dB impedance bandwidth.

It is worth mentioning that like the other microwave phase shifters, the proposed phase shifter has the properties of the microwave band-pass filter. One of the important characteristics of the filters is that its pass band should be accompanied with a linear phase response [24,25,26,27,28], and the proposed phase shifter meets this condition properly.

Table 2 gives a comparison between the proposed stub-loaded phase shifter and some other important designs. The dimensions of each design are normalized to the wavelength ($\lambda_{o}$) corresponding to the first resonant frequency. It is clear that this work has a balance between the dimensions and the phase difference BW, as well as the presence of the computer-based modeling method.

## 7. Conclusions

The inaccuracy in the conventional modeling phase shifters has been solved in this work by proposing a new MATLAB code that gives the exact modeling equations. The proposed code provides computer-based modeling for the 90° stub-loaded phase shifter. The model is applied on two-, three-, four-, and five-stub phase shifters. The results of the modeling equations of the three-stub wideband 90° stub-loaded phase shifter is compared with the CST microwave studio results and experimental results to verify the accuracy of the proposed model. It was found that the results of the proposed model almost perfectly coincide with the simulation and experimental results with −10 dB bandwidth equal to 90% (3.1–8 GHz), $\left(90{}^{\circ}\mp 5{}^{\circ}\right)$ phase difference bandwidth equal to 100% (2.8–8.3 GHz), and insertion loss within 0.1 dB. It is clear that the novelty of this work resides in providing the exact modeling equations for any arbitrary stub-loaded phase shifter directly, without delving in the mathematical derivations manually. As a future work, the proposed MATLAB code will be developed to consider the strong coupling effect of the designed phase shifter.

## Figures and Tables

**Table 1:** Comparison between the modeled equation outcomes with the CST microwave studio results and the measured results.

	CST Simulation	Measurement	Modeled Equations
−10 dB impedance BW (%)	90.01%	90.01%	90%
Insertion loss (dB) @ 5.5 GHz	0.138	0.156	0
$\left(90{}^{\circ}\mp 5{}^{\circ}\right)$ phase difference BW (%)	100%	94%	100%

**Table 2:** Comparison between the proposed phase shifter and other important designs, where $\lambda_{o}$ corresponds to the first resonant frequency.

Ref.	Dimensions	Phase Difference BW (%)	Modeling Method
[10]	$0.77\lambda_{o}\times 0.3\lambda_{o}\times 0.01\lambda_{o}$	102	Conventional Derivation
[13]	$0.32\lambda_{o}\times 0.22\lambda_{o}\times 0.01\lambda_{o}$	59	Conventional Derivation
[14]	$0.46\lambda_{o}\times 0.31\lambda_{o}\times 0.01\lambda_{o}$	64	Conventional Derivation
[16]	$0.69\lambda_{o}\times 0.46\lambda_{o}\times 0.014\lambda_{o}$	59	Conventional Derivation
[17]	$0.52\lambda_{o}\times 0.42\lambda_{o}\times 0.01\lambda_{o}$	77	--
This work	$0.53\lambda_{o}\times 0.4\lambda_{o}\times 0.02\lambda_{o}$	100%	Computer-Based

## Data Availability

Not applicable.

## Appendix A

The download link of the proposed MATLAB code is:

https://drive.google.com/file/d/1u78UyVAuNLfIHOcrO5wlptoHVuQmS034/view?usp=sharing (accessed on 1 September 2023).

Another public repository (GitHub) download link of the proposed MATLAB code is:

https://github.com/Abdulghafor1/Matlab-codes/blob/main/General_Code_Stub_Loaded_PS.m (accessed on 1 September 2023).
